# Efficacy of selenium supplementation for mild-to-moderate Graves’ ophthalmopathy in a selenium-sufficient area (SeGOSS trial): study protocol for a phase III, multicenter, open-label, randomized, controlled intervention trial

**DOI:** 10.1186/s13063-023-07282-4

**Published:** 2023-04-14

**Authors:** Chae Won Chung, Kyong Yeun Jung, Eun Hye Jung, Min Joung Lee, Young Joo Park, Jeong Kyu Lee, Hwa Young Ahn, Sun Wook Cho

**Affiliations:** 1 Department of Internal Medicine, College of Medicine, Seoul National University, 101, Daehak-Ro, Jongro-Gu, Seoul, 03080 Republic of Korea; 2 Department of Internal Medicine, Nowon Eulji Medical Center, Eulji University, Seoul, Republic of Korea; 3 Department of Ophthalmology, Nowon Eulji Medical Center, Eulji University, Seoul, Republic of Korea; 4 Department of Ophthalmology, Hallym University Sacred Heart Hospital, Anyang, Republic of Korea; 5 Department of Molecular Medicine and Biopharmaceutical Sciences, Graduate School of Convergence Science and Technology, Seoul National University, Seoul, Republic of Korea; 6 Department of Internal Medicine, Seoul National University Hospital, Seoul, Republic of Korea; 7 Department of Ophthalmology, College of Medicine, Chung-Ang University, Seoul, Republic of Korea; 8 Department of Internal Medicine, College of Medicine, Chung-Ang University, 102, Heukseok-Ro, Dongjak-Gu, Seoul, 06973 Republic of Korea

**Keywords:** Graves’ ophthalmopathy (GO), Selenium, Graves’ disease, Quality of life

## Abstract

**Background:**

The therapeutic effect of selenium has been demonstrated in mild Graves’ ophthalmopathy (GO) in a European region where selenium status is suboptimal. However, there is a lack of evidence to support selenium use in selenium-sufficient areas. The aim of this study is to evaluate the therapeutic effect of selenium in mild-to-moderate GO in selenium-sufficient South Korea.

**Methods:**

The SeGOSS trial is a multicenter, prospective, randomized, open-label trial in South Korea. Eighty-four patients aged 19 years or older with mild-to-moderate GO will be randomized to receive either vitamin B complex alone or vitamin B complex with selenium for 6 months with three monthly follow-up visits. The primary outcome is comparison of the improvement in quality of life at 6 months from baseline between the control and selenium groups. The secondary outcomes are intergroup differences in changes in quality of life at 3 months, clinical activity of GO at 3 and 6 months, thyroid autoantibody titers at 3 and 6 months, and the response rate at 3 and 6 months from baseline. Quality of life will be measured by questionnaire for patients with GO, and the clinical activity of GO will be evaluated by the clinical activity score (CAS). A positive response is defined as either changes in the CAS < 0 or the changes in the GO-QOL score ≥ 6.

**Discussion:**

The SeGOSS study will evaluate the therapeutic potential of selenium for mild-to-moderate GO in a selenium-sufficient area and provide support in tailoring better treatment for GO.

**Trial registration:**

KCT0004040. Retrospectively registered on 5 June 2019. https://cris.nih.go.kr/cris/search/detailSearch.do/14160.

**Supplementary Information:**

The online version contains supplementary material available at 10.1186/s13063-023-07282-4.

## Background

Graves’ ophthalmopathy (GO) is a complex immune-mediated inflammatory disorder of the orbit and periorbital tissues characterized by proptosis, upper eyelid retraction, ocular dryness, and periorbital swelling and/or redness. The annual incidence is 1.9 cases per 10,000 population [1], and approximately 50% of patients with Graves’ disease have ocular involvement [1, 2]. Treatment of GO is stratified according to the stage of the disease. Moderate-to-severe GO with active inflammation can be treated with glucocorticoids and/or orbital irradiation [2, 3], whereas patients with mild GO are generally managed with a wait-and-see strategy and conservative local therapies including artificial tears, ointments, and prisms. However, most patients with mild GO also have substantial problems in their quality of life [4, 5]. In addition, 15% of them are known to experience disease progression [6]. Therefore, it is reasonable to suggest that mild-to-moderate GO should be treated specifically based on its pathophysiology.

Selenium is an essential micronutrient that is abundant throughout the body, especially in the thyroid. In the form of selenocysteine, which is incorporated into proteins (mainly enzymes), selenium participates in thyroid hormone synthesis and exerts antioxidative effects [7, 8]. In healthy individuals, the reactive oxygen species (ROS) generated during thyroid hormone synthesis are cleared by glutathione peroxidases, which are selenoproteins, but there could be unmet needs for selenium in patients with autoimmune thyroid disorders whose levels of ROS are abnormally elevated [7, 9–11]. Based on those hypotheses, several studies have provided selenium supplementation for patients with Hashimoto’s thyroiditis and have reported decreases in thyroid peroxidase antibody (anti-TPO Ab) titers and normalization of the thyroid parenchyma on ultrasonography [12–15]. For patients with mild GO, a previous randomized double-blinded placebo-controlled trial showed that treatment of 6 months of selenium supplementation significantly reduced disease progression and improved quality of life [16]. Subsequently, selenium has been highlighted as an affordable treatment option for mild GO rather than the wait-and-see strategy [17, 18]. Considering the pivotal role of ROS in the pathogenesis of GO, it is reasonable that selenium may alleviate the course of disease in selenium-deficient areas [16]. However, there has been lack of evidence about the effects of selenium supplementation for mild-to-moderate GO in selenium-sufficient areas, such as South Korea, Australia, and North America. Notably, selenium is not recommended as a major treatment option for mild GO by the American Thyroid Association members in North America [19].

With regard to alternative therapeutics, vitamin B complex is introduced in this study. Deficiencies of B vitamins, including thiamine, folate, and cobalamin, are common in patients with autoimmune thyroid disease [20–23], and traditionally many patients have been supplemented with B vitamins. Especially, patients with Graves’ disease showed high prevalence of vitamin B deficiency, and the autoantibody titer was negatively associated with vitamin B12 [21, 24, 25]. From a mechanical point of view, B vitamins regulate immune cell function by supporting essential cofactors related to energy metabolism [26], especially supports NK-T and CD8 + T cells [27, 28]. Additionally, vitamin B12 has antioxidant properties that scavenge ROS [29], which is one of the pivotal pathophysiologic mechanisms of autoimmune thyroid disease including Graves’ disease [30–33].

In this multicenter, randomized, open-label clinical trial, we aim to evaluate whether selenium and/or vitamin B12 have beneficial effects on mild-to-moderate GO in a selenium-sufficient area (the SeGOSS trial).

## Methods

### Objectives

The primary objective of this study is to compare the efficacy of interventions to improve the quality of life in patients with GO after 6 months of each treatment with either (1) vitamin B complex or (2) vitamin B complex with selenium. The secondary objective involves an earlier comparison and assessment of GO progression marker (clinical activity score (CAS) and autoantibody titer) at 3 months.

### Trial design

This trial is a multicenter, prospective, randomized, open-label superiority trial conducted in South Korea (version 4.0; date 16 March 2021) to assess the clinical outcome of selenium supplement in patients with mild to moderate GO. After screening patients for mild-to-moderate GO, the participants will be randomly allocated into two arms: vitamin B complex (control group) or vitamin B complex with selenium (selenium group). Treatment will be continued for 6 months, and efficacy assessment will be performed at 3 and 6 months after the first treatment.

The trial was registered with the Clinical Research Information Service of the Republic of Korea (https://cris.nih.go.kr/cris/search/detailSearch.do/14160), which belongs to the World Health Organization International Clinical Trials Registry Platform, on 5 June 2019. The trial registration number is KCT0004040. This study protocol was developed following the Standard Protocol Items: Recommendations for Interventional Trials (SPIRIT) (see Additional file 1).

The recruitment period is from May 2019 to March 2023, and the follow-up data will be collected until September 2023.

### Study setting

The study participants will be recruited from and assessed at Seoul National University Hospital (Seoul, South Korea), Chungang University Hospital (Seoul, South Korea), Nowon Eulji Hospital (Seoul, South Korea), and Hallym University Sacred Heart Hospital (Gyeonggi-do, South Korea). All four centers are high-volume centers and will recruit participants at outpatient clinic following the same strategy for recruiting and assessment of quality of life and GO progression as indicated in the study protocol.

### Study population

The study population will consist of patients with mild-to-moderate GO who have provided written informed consent. After screening for eligibility, a total of 84 patients will participate in this study. Forty-two patients will be randomly allocated to receive either vitamin B complex or vitamin B complex with selenium.

### Eligibility criteria

The final participants will be selected after screening for the following eligibility criteria (Table 1).

**Table 1:** Eligibility criteria

Inclusion criteria	Exclusion criteria
1. Age 19–75 years of either gender 2. Patients with mild-to-moderate GO with one of the following: - Confirmation of mild-to-moderate GO by a professional ophthalmologist - NO SPECS class 2a or 2b (mild or moderate involvement of soft tissue) [61] - Proptosis of 22 mm or less - No treatment for GO other than symptom control since diagnosis 3. Patients with Graves’ disease with normal thyroid function as indicated: - Patients maintaining euthyroid status for at least 2 months with ATDs - Patients with maintenance of euthyroid status from 6 months after RAI treatment - If hypothyroidism occurred after Grave’ disease treatment including with ATDs, RAI, or surgery, patients who have euthyroid status under conditions of thyroid hormone replacement - Patients who have been diagnosed with GO by an ophthalmologist but have not yet developed thyrotoxicosis and who are positive for anti-thyroid receptor antibodies 4. Patients who have voluntarily signed the written consent form	1. Patients with severe GO who meet at least one of the following criteria: - NO SPECS class 2C (marked involvement of soft tissue) - Proptosis greater than 22 mm - Diplopia or ocular torticollis in a resting position - Optic neuropathy - Confirmation of severe GO by an ophthalmologist 2. Patients who have been treated with a high dose of steroids or any dose of selenium within 3 months: - High-dose steroid treatment includes oral methylprednisolone more than 1 g per day or equivalent and intravenous steroids 3. Patients with contraindications for selenium supplementation meeting at least one of the following criteria: - Hypersensitivity to selenium or a component of the pill - Selenium toxicity - Hereditary fructose intolerance, glucose-galactose malabsorption, or deficiency of sucrase or isomaltase 4. Patients with comorbidities or active disease under treatment 5. Pregnant or lactating women

Inclusion and exclusion criteria for the SeGOSS study

*Abbreviations*: *GO* Graves’ ophthalmopathy, *ATD* antithyroid drug, *RAI* radioactive iodine

### Sample size

The sample size of this study is based on a previous randomized clinical trial of selenium supplementation in individuals with mild GO in Europe. According to a previous randomized clinical trial of mild GO (placebo, selenium, and pentoxifylline administration in each treatment group) that measured the GO Quality of Life Questionnaire (GO-QOL) [5], we hypothesized that the baseline GO-QOL score in mild GO would be 80 ± 16 [16]. To show a significant increase, we assumed a 12% increase in the GO-QOL score of the experimental group compared with the control group. We calculated the sample size with 1:1 allocation with at least 80% power and an alpha error confidence level of 5%. Using the sample size calculating formula [34], each group would require 40 patients. Considering a 5% drop-out rate, this trial would require a total of 84 patients to be randomized (42 of whom will be included each group).

### Recruitment and initial assessment

After identification of a potential participant meeting the eligibility criteria, the researchers will verbally provide information about the trial at the outpatient clinic. If the patient agrees to participate, the researcher will collect a written informed consent form from the patient. The patient will be informed the voluntary withdrawal from the trial is possible at any time and that withdrawal will not affect his or her treatment at all.

The initial assessment will include complete history taking, ophthalmic examination performed by an ophthalmologist, assessment of GO activity (GO-QOL, CAS, orbital computed tomography (CT), and frontal view photo of the eyes), collection of anthropometric data (body weight, height, blood pressure, and pulse rate), assessment of hematologic and biochemical analyses (complete blood count (CBC), aspartate aminotransferase (AST), alanine aminotransferase (ALT), serum creatinine and blood urea nitrogen (BUN), fasting glucose, lipid profile, free T4 and/or free T3, thyroid-stimulating hormone (TSH), anti-thyroid peroxidase antibody (TPO Ab), thyrotropin-binding inhibitor immunoglobulin (TBII), and selenium), and thyroid ultrasound. Participants who still meet the inclusion criteria will be formally included in the trial.

### Randomization

**Fig. 1:**
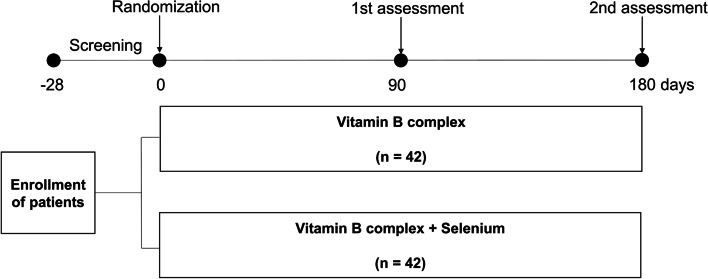
Schematic description of the study design. After screening, the enrolled patients will be randomized to receive either vitamin B complex only (control group) or vitamin B complex + selenium (selenium group) for 6 months. Assessments including the quality of life and clinical activity of GO evaluation, thyroid function testing, and thyroid autoantibody titers will be performed at 3 and 6 months

Eighty-four patients will be randomly allocated to each arm in a 1:1 ratio. Block randomization with block size of four will be used. The computerized randomization codes and sequence will be generalized and concealed by a statistician who is not associated with the conduct of study in each center. When a patient is enrolled in the study, each code from a block sequence will be allocated and the assigned group indicated in the sealed envelope will be supplied to each patient. Either vitamin B complex or vitamin B complex with selenium will be administered according to the code assigned by a clinical research coordinator (CRC) who is not associated with data analyses. Each allocation will be recorded and kept secure by the CRC and will be opened in the case of severe adverse events related to study drop-out. The recruitment and randomization of this trial is described in Fig. 1.

### Intervention

#### Control group

Vitamin B complex, which consists of vitamin B_1_ (24 mg), vitamin B_2_ (28 mg), niacin (15 mg), vitamin B_6_ (60 mg), folate (400 μg), vitamin B_12_ (96 μg), biotin (300 μg), and pantothenic acid (50 mg) will be given as one tablet daily during the 6-month trial. Each amount of the B vitamins is within the recommended level or does not exceed the tolerable upper intake level according to 2020 Nutrient Intake Standards for Koreans [35].

#### Selenium group

Vitamin B complex with selenium will be supplemented during the 6-month trial. The vitamin B complex tablet will be the same as that received by the control group. Selenium will be given as sodium selenite at a dose of 100 μg twice daily.

### Concomitant treatment

Treatment with antithyroid drugs (ATDs) including methimazole, carbimazole, and propylthiouracil will be allowed to be initiated or continued during the study. Local therapies for symptomatic treatment of mild GO including artificial tears, ointments, and prisms will also be allowed, while surgical interventions or periorbital injections with steroids or botulinum toxin will be prohibited during the study. Multi-vitamins or nutritional supplements, especially those acting as a functional reductase, such as vitamin C, will be prohibited during the study.

### Procedures and outcomes

The interventional treatment will be performed for 6 months, and patients will be monitored closely with anthropometric and cardiometabolic indicators, and laboratory examinations at 3 and 6 months. An overview of study visits and assessments per visit is summarized in Table 1.

An ophthalmic examination, CAS determination, and frontal photography of both eyes will be performed at every visit. An orbital CT scan will be performed only at baseline to confirm a diagnosis of mild-to-moderate GO. The same ophthalmologist or trained endocrinologist at each center will perform an ophthalmic examination including assessments of eyelid aperture size (palpebral fissure), exophthalmos, and extraocular muscle involvement (diplopia) at every visit. Eyelid aperture size will be measured in millimeters, and soft tissue involvement will be assessed following the GO clinical evaluation atlas [36]. The CAS is a grading system used to measure the severity of GO [37]. A score of more than 3 points indicates active inflammation adding 1 point for each sign or symptoms as follows: (1) spontaneous orbital pain, (2) gaze-evoked orbital pain, (3) eyelid swelling, (4) eyelid erythema, (5) conjunctival redness, (6) chemosis, and (7) inflammation of the caruncle or plica. Frontal photography of the eyes will be reviewed by an experienced ophthalmologist at a central laboratory to confirm the disease status of each patient with GO (Table 2). Upon completion of the screening test and written informed consent, participants underwent eye examination, anthropometric and cardiometabolic assessment, and blood sampling according to the schedule.

**Table 2:** Schedule of data collection during the follow-up period

	Screening	3 months	6 months
**Eligibility screening**	X		
**Informed consent**	X		
**Eye examination**			
Physical examination	X	X	X
GO-QOL questionnaire	X	X	X
CAS determination	X	X	X
Orbital CT (noncontract)	X		
Frontal photography of the eyes	X	X	X
**Anthropometric and cardiometabolic assessment**			
Height	X		
Weight	X	X	X
Blood pressure	X	X	X
Pulse rate	X	X	X
**Laboratory tests**			
Selenium	X		X
TSH, free T4 and/or free T3	X	X	X
TBII and anti-TPO Ab	X	X	X
CBC	X		X
AST and ALT	X		X
BUN and creatinine	X		X
Fasting glucose	X		X
Total and LDL cholesterol, triglycerides	X		X
Thyroid ultrasonography	X		X

*Abbreviations*: *GO-QOL* Graves’ Ophthalmopathy Quality of Life Questionnaire, *CAS* clinical activity score, *CT* computed tomography, *TSH* thyroid-stimulating hormone, *TBII* thyrotropin-binding inhibitor immunoglobulin, *anti-TPO Ab* thyroid peroxidase antibody, *CBC* complete blood count, *AST* aspartate aminotransferase *ALT* alanine aminotransferase, *BUN* blood urea nitrogen, *LDL* low-density lipoprotein

Quality of life will be assessed by the GO-QOL completed by participants at baseline, at 3 and 6 months, and at 6 months after termination of the trial. The GO-QOL created by Terwee et al. [38] is a useful modality to measure quality of life among patients with GO and has been validated in several studies performed in various regions [38–41]. Due to its high association with disease activity and treatment response [42–46], the European Group on Graves’ Orbitopathy has recommended the use of the GO-QOL as an evaluation tool in clinical trials [47]. The GO-GOL consists of two subscales: visual functioning and appearance. Each subscale includes eight questions, and 1 to 3 points are assigned for each question [see Additional file 2 for the detailed context in the both the English and Korean versions of the GO-QOL].

Anthropometric and cardiometabolic measurements including body weight, blood pressure, and pulse rate will be performed at every visit. Among blood tests, thyroid functions tests (TSH and free T4 or free T3) and thyroid autoantibody titers will be measured at every visit to detect Graves’ disease status. Hematologic (CBC) and biochemical analyses (AST/ALT, serum creatinine and BUN, fasting glucose, lipid profile, freeT4 and/or free T3, TSH, thyroid autoantibody, and selenium) will be performed at baseline and at 6 months. Thyroid function and chemistry tests, which are listed in Table 1, will be performed at each center in real time, while TBII and anti-TPO Ab titers and plasma selenium levels will be checked at the central laboratory in the end of the study. For plasma selenium analysis, fasting plasma samples will be stored at – 70 °C and measured at a central laboratory at the same time using inductively coupled plasma mass spectrometry (ICP-MS 7800, Agilent Technologies, Santa Clara, CA, USA).

To improve adherence to the protocol, the drug tablet return will be checked at every visit. Any adverse events will be determined, recorded, and followed at each visit. Furthermore, participants will be able to contact the CRC responsible for adverse event management at any time by phone and report other unintended effects of drug replacement.

This trial has primary and secondary outcomes as described below:

#### Primary outcome

1. Improvement in the GO-QOL score at 6 months after baseline treatment

#### Secondary outcome

1. Changes in the GO-QOL score at 3 months after baseline treatment
2. Changes in the CAS at 3 and 6 months after baseline treatment
3. Changes in TBII and anti-TPO Ab titers at 3 and 6 months after baseline treatment
4. Response rate at three and 6 months after baseline treatment. A positive response was defined as meeting one of the following criteria:
5. Change in the CAS < 0
6. Change in the GO-QOL score ≥ 6

### Withdrawal and drop-out

Participants will be able withdraw their consent at any time voluntarily and will not be required to justify their withdrawal. In patients with program violation or one of the following during the trial period, the research team will request withdrawal after considering the benefits and harms of participation in the clinical trial: (1) progression of GO that requires intravenous steroid or decompressive surgery; (2) occurrence or aggravation of concomitant disease or serious adverse event [48] preventing replacement of the trial drug; and (3) other reliable reason for which trial drug administration is harmful to the participant such as pregnancy after trial initiation. In all cases, the reason for withdrawal will be recorded in the eCRF and in the patient’s medical records. In case of withdrawal of a patient at his/her own request, the reason will be asked for as extensively as possible and documented. For patients with incomplete follow-up, time to last follow-up date will be used as the censoring time in the analysis of time-to-event data. Missing data of continuous outcomes over time will be handled via the multi-level approach making the implicit assumption that data are missing at random, thus not requiring any direct imputation of missing continuous data. The robustness of this assumption will be explored in sensitivity analyses by means of pattern mixture models assuming that data are not missing at random. Otherwise, no imputation of missing data will be conducted.

Participants will be educated frequently by the investigators to report their adverse events in a timely manner. The research team will perform detailed interviews, measurement of vital sign, and physical examinations at every visit and evaluate for any abnormalities related to trial medications, GO, or any other medications the participant is taking. If a serious adverse event occurs, the patient will be actively treated and followed up at each clinic after drop-out. There is no anticipated harm and compensation for trial participation.

### Data management and monitoring

Data including the date of agreement, anthropometric and cardiometabolic data, history of illness, physical examination findings, measurements, and results of analyses will be collected on computerized case report forms. The collection and storage of data will be compliant with the International Conference on Harmonization (ICH)—Good Clinical Practice (GCP) guidelines [49]. All the clinical and genetic information of participant will be selectively opened for only sub-, coordinating, and principal investigators. Data related to participant identification will not be recorded, and a separate identification number will be assigned. The data file will be locked with a password, and the hard copy of data will be stored in a double-locked locker. According to Article 15 of the Enforcement Rules of the Bioethics Act, the data of this study will be kept for 3 years or less from the time the research is completed and will be destroyed within 3 years.

The research subjects will complete a consent form for research on human specimens to specify the preservation period for the storage and disposal of human specimens collected from them, whether they are provided for other research purposes, and the personal information when provided. Human derivatives of research subjects who have not consented to secondary use within the preservation period shall be discarded after the preservation period. Human derivatives of research subjects who have agreed to secondary use within the preservation period can be provided for other research purposes within the preservation period, and after the preservation period, human derivatives will be discarded. If the research must be terminated abnormally, the human material will be transferred or discarded according to deliberation of institutional review board (IRB) in each center. We will manage human derivatives as specified in the standard operating procedure and discard human derivatives that have passed the retention period determined by the research subject.

The data safety monitoring board will consist of representatives from the IRB, including external experts in the field of endocrinology, ophthalmology, statistics, and pharmacology. The trial auditors will monitor subjects at least once a year to monitor the safety, consistency of data collection, whether the trial is implemented following the protocol, and need for early termination due to adequate benefit or severe harm.

The coordinating and steering committee is Seoul National University Hospital.

### Statistical methods

This trial is designed to compare the selenium group and control group. The primary and secondary outcomes will be analyzed in accordance with the intention to treat (ITT) and per protocol (PP) principle. For PP analysis, participants who drop out from the trial before 3 months or have severe adverse event will be excluded from the analysis. The last measurements from patients who withdraw from the study before trial termination and have data for the timepoint at 3 months will be included for comparisons only at 3 months.

The baseline characteristics will be examined by a descriptive analysis of questionnaire responses and laboratory findings. Student’s *t*-test will be used for normally distributed values, and the Mann–Whitney *U* test will be used for non-normally distributed ones. Categorical values will be examined using the chi-square test or the Fisher exact test.

To evaluate the improvement after treatment in each group, the change in GO-QOL score, CAS, thyroid function, and thyroid autoantibody titer will be analyzed by a paired-sign test within the control group and selenium group. Thyroid function will be evaluated in real time at each clinic with different measurement kits; thus, the analysis within each clinic will be performed subsequently.

The intergroup differences at 3 and 6 months in the GO-QOL score, CAS, thyroid function, and thyroid autoantibody titer will be analyzed on Student’s *t*-test. To compare the ratio of participants with improvement or deterioration between the control group and selenium group, the chi-square test will be used. An age- and sex-adjusted mixed-effects model considering repeated measures and time term will be used for comparisons from baseline to 3 and 6 months after treatment between groups. A *P* value of less than 0.05 was considered to indicate statistical significance.

A subgroup analysis of the primary endpoint will involve the paired-sign test and Student’s *t*-test approach to estimate improvement of GO-QOL and CAS in the active GO patients defined as a CAS 2 or higher. There will be no interim analysis.

### Ethics and dissemination

The study protocol has been approved by the ethics committees of each center: Seoul National University Hospital (H-1812–026-991), Chungang University Hospital (1912–002-358), Nowon Eulji Hospital (2020–07-013), and Hallym University Sacred Heart Hospital (2020–07-007–001). The ICH-GCP will be strictly followed during trial. The study protocol has been developed in accordance with the Helsinki Declaration, and participants who provide written informed consent will be selectively included in the study. Any problems associated with the trials such as serious adverse events, revision of the protocol, and termination or early termination of the trial will be reported in a timely manner to the ethics committee of each clinic. Before recruited to the trial, every participant will be closely informed about the objective and the content of the trial and complete the written informed consent form. If the consent form is revised, the investigators will inform the participants about the changes, abrogate their prior consent, and obtain new written consent. All participants will receive orientation to the trial by trained research nurses and provide written informed consent.

The results of this study will be published in a peer-reviewed journal and/or at a national or international conference. The researchers involved in design, collection and analysis of data, writing, and discussion are listed as authors. The dataset generated during the study will be available from the corresponding author upon responsible request.

Authorship eligibility of this trial is based on the following criteria (ICMJE): (1) substantial contributions to the conception or design of the work or the acquisition, analysis, or interpretation of data for the work; (2) drafting the work or revising it critically for important intellectual content; (3) final approval of the version to be published; and (4) agreement to be accountable for all aspects of the work in ensuring that questions related to the accuracy or integrity of any part of the work are appropriately investigated and resolved. There is no intended use of professional writers.

## Discussion

Several studies have attempted to clarify the relationship between selenium and autoimmune thyroid diseases. In Hashimoto’s thyroiditis, 200 μg of sodium selenite or selenomethionine per day for 3 and 6 months was found to alleviate auto-TPO Ab and anti-thyroglobulin antibody titers and improve thyroid echogenicity [12, 50]. For maximal glutathione peroxidase activity, Turker et al. [51] showed that at least 200 μg of selenomethionine per day was required, and the autoantibody titer was increased in patients taking 100 μg of selenium per day. In Graves’ disease, selenium not only normalized the TSH receptor autoantibody titer but also enabled more rapid attainment of euthyroid status in patients receiving selenium supplementation and ATDs compared to those receiving ATDs alone [52, 53]. To achieve a comprehensive understanding of the role of selenium in autoimmune thyroid disease, the Chronic Autoimmune Thyroiditis Quality of Life Selenium Trial (CATALST) and GRAves’ disease Selenium Supplementation (GRASS) trials were conducted by the same group of investigators, and the outcomes have yet to be published [54, 55].

Regarding the pathophysiology of GO, ROS production seems to play a major role in disease onset and progression [56, 57]. As alternative therapeutics, vitamin B complex is provided to participants in anticipation of the antioxidant effect [29]. Low selenium levels have been reported in GO [58], and selenium replacement was reported to ameliorate quality of life and delay disease progression in patients with mild GO [16]. Based on data from adults in selenium-deficient areas, the 2022 European Thyroid Association recommended selenium support in patients with mild pediatric Graves’ disease with orbitopathy [59]. However, Owji et al. reported comparable levels of serum selenium between patients with GO and healthy individuals [60]. In addition, there is a paucity of data regarding the association between selenium and GO in selenium-rich areas. Therefore, the SeGOSS study is expected to yield helpful findings for making decisions in the clinical field in selenium-rich areas such as South Korea.

## Trial status

This trial protocol is Version 4.0, 16 March 2021. Recruitment began on 14 May 2019 and is anticipated to be completed by 31 March 2023.

## Supplementary Information

**Additional file 1.** SPIRIT 2013 Checklist. Recommended items to address in a clinical trial protocol and related documents.

**Additional file 2.** Graves’ Ophthalmopathy Quality of Life Questionnaire (GO-QOL). Detailed items of GO-QOL questionnaire.

## Data Availability

Restrictions are applied to the availability of data generated or analyzed during this study to preserve patient confidentiality. The corresponding author will detail the restrictions and any conditions under which access to some data may be provided upon request.

## Abbreviations

GO: Graves’ ophthalmopathy
ROS: Reactive oxygen species
Anti-TPO Ab: Thyroid peroxidase antibody
CAS: Clinical activity score
SPIRIT: Standard protocol items: recommendations for interventional trials
GO-QOL: Graves’ Ophthalmopathy Quality of Life Questionnaire
CT: Computed tomography
CBC: Complete blood count
AST: Aspartate aminotransferase
ALT: Alanine aminotransferase
BUN: Blood urea nitrogen
TSH: Thyroid-stimulating hormone
TBII: Thyrotropin-binding inhibitor immunoglobulin
CRC: Clinical research coordinator
ATD: Antithyroid drug
ICP-MS: Inductively coupled plasma mass spectrometry
IRB: Institutional review board
ICH: International Conference on Harmonization
GCP: Good clinical practice
CATALYST: Chronic Autoimmune Thyroiditis Quality of Life Selenium Trial
GRASS: GRAves’ Disease Selenium Supplementation
