# Supplementary material for: Efficacy of selenium supplementation for mild-to-moderate Graves’ ophthalmopathy in a selenium-sufficient area (SeGOSS trial): study protocol for a phase III, multicenter, open-label, randomized, controlled intervention trial
Source: Trials. 2023 Apr 14;24:272. doi: 10.1186/s13063-023-07282-4 (PMC10103450; doi:10.1186/s13063-023-07282-4)
Supplement: Supplementary file 2 — Additional file 2. Graves’ Ophthalmopathy Quality of Life Questionnaire (GO-QOL). Detailed items of GO-QOL questionnaire. [file 13063_2023_7282_MOESM2_ESM.pdf]

Graves' ophthalmopathy quality of life questionnaire (GO-QOL)

Subscale: Visual Functioning (VF)

During the past week, to what extend were you limited in carrying out the following activities, because of

	Yes, seriously **(1 point)**	Yes, a little **(2 point)**	No, not at all **(3 point)**
1. Bicycling<br>(never learned to ride a bike:_________)			
2. Driving<br>(no driver's licence: __________)			
3. Moving around the house			
4. Walking outdoors			
5. Reading			
6. Watching TV			
7. Hobby or pastime, e.g_______________			
8. During the past week, did you feel hindered from something that you wanted to do because of your thyroid eye disease?			

Subscale: Appearance

The following questions deal with your thyroid eye disease in general:

	Yes, very much **(1 point)**	Yes, a little **(2 point)**	No, not at all **(3 point)**
9. Do you feel that your appearance has changed because of your thyroid eye disease?			
10. Do you feel that you are stared at in the streets because of your thyroid eye disease?			
11. Do you feel that people react unpleasantly because of your thyroid eye disease?			
12. Do you feel that your thyroid eye disease has an influence on your self-confidence?			
13. Do you feel socially isolated because of your thyroid eye disease?			
14. Do you feel that your thyroid eye disease has an influence on making friends?			
15. Do you feel that you appear less often on photos than before you had your thyroid eye disease?			
16. Do you try to mask changes in appearance caused by your thyroid eye disease?			

갑상선안병증 환자의 '삶의 질' 설문지

등록번호______________

지난 1주일간의 생활 속에서 갑상선안병증으로 인해 다음과 같은 활동들이 얼마나 제한을 받았는지 생각해 보고, 알맞은 칸에 표시(V)해 주세요.

	심각한 제한이 있다	약간 제한이 있다	전혀 제한이 없다	타는 법을 배우지 않았다
1. 자전거 타기				
2. 운전				
3. 실내에서 돌아다니기				
4. 실외에서 돌아다니기				
5. 책, 신문 읽기				
6. TV를 보기				
7. 취미 활동, 여가생활				
8. 하고자 하는 일을 할 때 갑상선안병증 때문에 방해 받습니까?				

다음은 갑상선안병증과 관련된 전반적인 질문입니다. 알맞은 칸에 표시해 주세요.

	심각한 제한이 있다	약간 제한이 있다	전혀 제한이 없다
9. 갑상선안병증으로 당신의 외모가 바뀌었다고 생각하나요?			
10. 갑상선안병증 때문에 길에서 다른 사람이 당신을 쳐다본다고 생각하나요?			
11. 갑상선안병증 때문에 사람들이 당신을 불쾌하게 대한다고 생각하나요?			
12. 갑상선안병증이 당신의 자존감에 영향을 미치나요?			
13. 갑상선안병증 때문에 사회적으로 격리되었다고 생각하나요?			
14. 갑상선안병증이 친구를 사귀는 데 영향을 미친다고 생각하나요?			
15. 갑상선안병증으로 당신은 사진을 찍는 게 꺼려지시나요?			
16. 갑상선안병증으로 인해 생긴 외모의 변화를 화장, 안경, 모자 등으로 가리고자 하시나요?			
